# Safety and Immunogenicity of a Third Dose of SARS-CoV-2 mRNA Vaccine – An Interim Analysis

**DOI:** 10.21203/rs.3.rs-1222037/v1

**Published:** 2022-05-03

**Authors:** Evan Anderson, Lisa Jackson, Nadine Rouphael, Alicia Widge, David Montefiori, Nicole Doria-Rose, Mehul Suthar, Kristen Cohen, Sarah O’Connell, Mat Makowski, Mamodikoe Makhene, Wendy Buchanan, Paul Spearman, C. Buddy Creech, Sijy O’Dell, Stephen Schmidt, Brett Leav, Hamilton Bennett, Rolando Pajon, Christine Posavad, John Hural, John Beigel, Jim Albert, Kuleni Abebe, Amanda Eaton, Christina Rostad, Paulina Rebolledo, Satoshi Kamidani, Daniel Graciaa, Rhea Coler, Adrian McDermott, Julie Ledgerwood, John Mascola, Stephen DeRosa, Kathleen Neuzil, M. Juliana McElrath, Paul Roberts

**Affiliations:** Kaiser Permanente Washington Health Research Institute; Emory University School of Medicine; National Institute of Allergy and Infectious Diseases/Vaccine Research Center; Duke; NIH; Emory University School of Medicine; Fred Hutchinson Cancer Research Center; Vaccine Research Center (VRC), National Institute of Allergy and Infectious Diseases (NIAID), NIH,; The Emmes Company, LLC; Division of Microbiology and Infectious Diseases, National Institute of Allergy and Infectious Diseases (NIAID), National Institutes of Health (NIH); Division of Microbiology and Infectious Diseases, National Institute of Allergy and Infectious Diseases (NIAID), National Institutes of Health (NIH); Cincinnati Children’s Hospital; Vanderbilt University School of Medicine; National Institutes of Health; NIH; Moderna, Inc.; Moderna, Inc.; Moderna, Inc.; Department of Laboratory Medicine and Pathology, University of Washington; Fred Hutchinson Cancer Research Center; Leidos Biomedical Research Inc.; The Emmes Company, LLC; FHI360; Duke Human Vaccine Institute; Emory University School of Medicine; Department of Medicine, Emory University School of Medicine; Center for Childhood Infections and Vaccines (CCIV) of Children’s Healthcare of Atlanta and Emory University Department of Pediatrics; Department of Medicine, Emory University School of Medicine; Center for Global Infectious Disease Research (CGIDR), Seattle Children’s Research Institute; National Institutes of Health; Vaccine Research Center - NIAID/NIH; National Institute of Allergy and Infectious Diseases; Fred Hutchinson Cancer Research Center and the University of Washington; U Maryland; Fred Hutchinson Cancer Research Center; Division of Microbiology and Infectious Diseases, National Institute of Allergy and Infectious Diseases (NIAID), National Institutes of Health (NIH)

## Abstract

Waning immunity after two SARS-CoV-2 mRNA vaccinations and the emergence of variants precipitated the need for a third dose of vaccine. We evaluated early safety and immunogenicity after a third mRNA vaccination in adults who received the mRNA-1273 primary series in the Phase 1 trial approximately 9 to 10 months earlier. The booster vaccine formulations included 100 mcg of mRNA-1273, 50 mcg of mRNA-1273.351 that encodes Beta variant spike protein, and bivalent vaccine of 25 mcg each of mRNA-1273 and mRNA-1273.351. A third dose of mRNA vaccine appeared safe with acceptable reactogenicity. Vaccination induced rapid increases in binding and neutralizing antibody titers to D614G, Beta, and Delta variants that were similar or greater than peak responses after the second dose. Spike-specific CD4+ and CD8+ T cells increased to similar levels as after the second dose. A third mRNA vaccination was well tolerated and generated robust humoral and T cell responses.

ClinicalTrials.gov numbers NCT04283461 (mRNA-1273 Phase 1) and NCT04785144 (mRNA-1273.351 Phase 1)

## Introduction

The mRNA-1273 SARS-CoV-2 vaccine, administered as a primary series of two 100 mcg doses 28 days apart, demonstrated 94% efficacy against COVID-19 illness in the pivotal Phase 3 efficacy trial,^1^ and was granted Emergency Use Authorization (EUA) by the US Food and Drug Administration (FDA) in December 2020. mRNA-1273 encodes for a prefusion stabilized full-length spike (S-2P) glycoprotein of the prototype SARS-CoV-2 Wuhan-Hu-1 isolate^2^ and induces robust neutralizing antibody responses to that strain and to the D614G variant,^3–5^ which was the predominant circulating SARS-CoV-2 variant for most of 2020.

Vaccine-induced neutralizing antibody responses against emerging variants, such as Beta (B.1.351) and Delta (B.1.617.2), are lower than against D614G, particularly for Beta.^6–10^ Assessment of serum samples obtained at six months after the two-dose mRNA-1273 primary series from participants in the Phase 1 mRNA-1273 trial indicated that neutralizing antibody titers waned with similar decay curves for D614G, Beta, and Delta.^9,11^ In response to the emergence of Beta, Moderna developed the mRNA-1273.351 vaccine that encodes for the stabilized full-length Beta S-2P.

Breakthrough infections including severe infections and hospitalizations are occurring in previously vaccinated individuals.^12,13^ While a third dose of COVID-19 mRNA vaccine directed against the prototype strain (Wuhan-Hu-1) is recommended, studies of a third dose of prototype versus a variant COVID-19 mRNA vaccine and a bivalent approach combining prototype and variant COVID-19 mRNA vaccines, are needed to determine the optimal third dose approach.

We evaluated the safety and immunogenicity of three booster vaccine formulations given eight to twelve months after a two-dose mRNA-1273 primary series in the dose-finding Moderna mRNA-1273 Phase I study.^3,4,9,11,14^ Here we report interim safety and immunogenicity after participants were boosted with a monovalent prototype boost of 100 mcg of mRNA-1273 (monovalent prototype), a monovalent variant boost of 50 mcg of mRNA-1273.351 (monovalent variant), or a bivalent boost of 25 mcg of mRNA-1273.351 and 25 mcg of mRNA-1273 (bivalent).

## Results

### Participants

**Figure 1:**
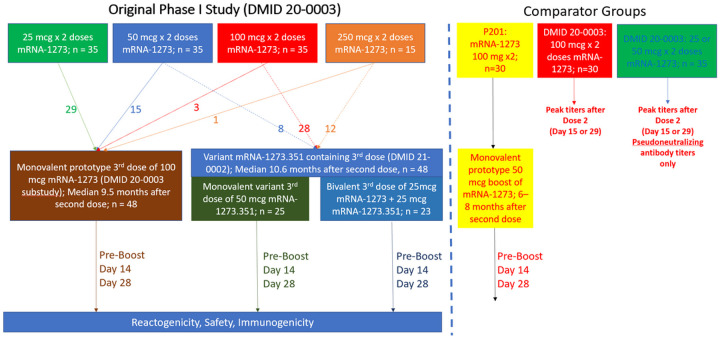
Enrollment and Timing of Samples Obtained for Participants and Comparators in this Study. The original Phase I mRNA-1273 study enrolled subjects ≥18 years of age and administered two doses of mRNA-1273 across 4 different dose levels (25, 50, 100, and 250 mcg). Those that had received any dose level were allowed to receive a single 100 mcg third dose (monovalent prototype vaccine group). Those that had received 50, 100, or 250 mcg doses were allowed to join a new study in which they were randomized to a single 50 mcg dose of monovalent variant vaccine (mRNA-1273.351) or a single dose of bivalent vaccine (25 mcg of mRNA-1273, 25 mcg mRNA-1273.351). For comparison, 30 adults that had received an initial 100 mcg of mRNA-1273 in the Phase 2 study followed by a 50 mcg third dose were included (P201). Peak responses observed at 2 or 4 weeks after a second dose of mRNA-1273 in those that had enrolled in the original Phase I study were used for comparison.

We evaluated three mRNA booster vaccines in participants who received their two-dose primary series as part of the mRNA-1273 Phase 1 dose-ranging trial (NCT04283461, DMID 20–0003) between March and June 2020.^3,4,9,11,14^ The phase 1 trial participants were recruited into three age-group strata (18–55 years, 56–70 years, and ≥71 years), and received a primary series of two mRNA-1273 vaccinations 28 days apart at doses of 25, 50, 100, or 250 mcg (Figure 1).

The monovalent prototype 100 mcg mRNA-1273 booster vaccine was evaluated in a substudy of the phase 1 trial and included any participant (any primary series dose) who chose to participate. The two variant-containing booster vaccines were evaluated in a new Phase 1 study (NCT04785144, DMID 21–0002) available to 20–0003 participants in the 50, 100, or 250 mcg dose groups who chose to leave the DMID 20–0003 phase 1 study and join the new trial. Participants in DMID 21–0002 were randomized 1:1 to receive a third mRNA vaccination in the Monovalent variant or Bivalent groups (Figure 1).

The characteristics of participants are shown in Table 1. The monovalent prototype group included 48 participants, of which 29, 15, 3, and 1 had received a 25, 50, 100, and 250 mcg mRNA-1273 primary series, respectively (Figure 1). The monovalent variant and bivalent groups also included 48 total participants, of which 8, 28, and 12 had received a 50, 100, or 250 mcg dose primary series, respectively. Participants in the monovalent prototype group tended to be older and had received their third dose at a median of 9.5 months (IQR 8.8, 10.4) after the second vaccination, while the monovalent variant and bivalent groups received their third dose 10.6 (IQR 10.0, 11.2) months after the second vaccination.

### Vaccine Reactogenicity and Safety

**Figure 2:**
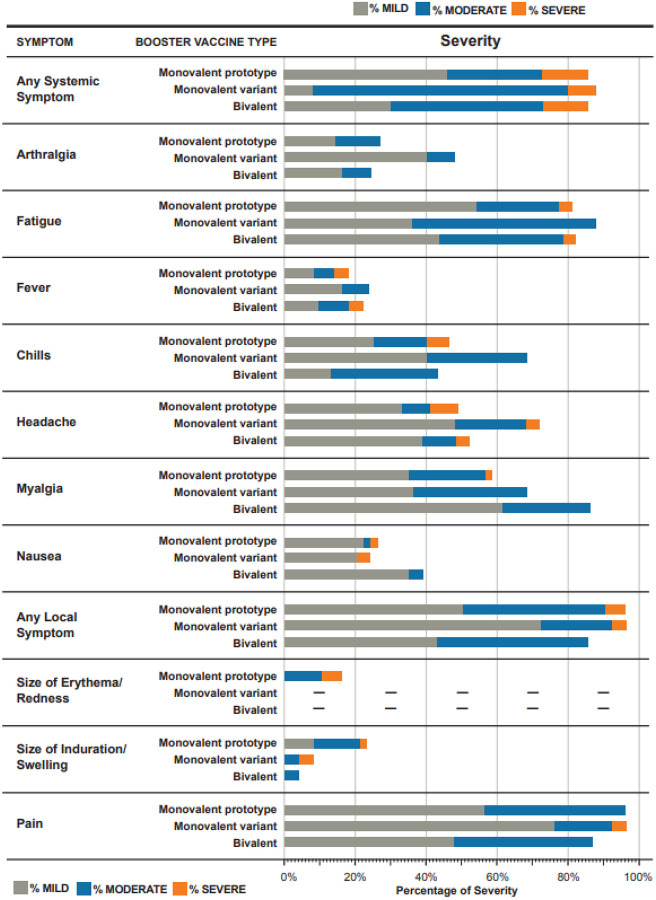
Reactogenicity in Participants After a Third Dose by Group. Solicited adverse events in the week after receiving a third dose of mRNA vaccine by study group: Monovalent prototype (50 mcg of mRNA-1273), Monovalent variant (50 mcg of mRNA-1273.351), and Bivalent (25 mcg of mRNA-1273, 25 mcg of mRNA-1273.351). Reactogenicity was generally similar across the study groups. Erythema/redness was not observed by participants in the monovalent variant and bivalent groups.

Among the solicited local AEs, pain at the injection site was most common and observed in 87–96% of participants across groups. In the monovalent prototype group, solicited systemic AEs were common (fatigue most commonly) (Figure 2), with 41 (85%) participants reporting at least one solicited systemic AE and 6 (13%) reporting at least one severe solicited systemic AE (chills and headache most commonly). In the monovalent variant group, 22 of the 25 (88%) participants reported at least one solicited systemic AE (fatigue most commonly). Of those with solicited systemic AEs, 2 (8%) reported a severe event (headache and nausea). In the bivalent group, 20 of the 23 (87%) participants reported at least one solicited systemic AE (fatigue and myalgia most commonly). Of these solicited systemic AEs, 3 (13%) reported a severe event (fever [peak 39.1°C], fatigue, and headache).

Unsolicited adverse events reported within 28 days were usually mild to moderate in severity (**Supplementary Results and Tables S2 – S6**). Severe related unsolicited adverse events in the monovalent variant group included a participant with vomiting and another participant with medically-attended generalized pruritic rash beginning 11 days after vaccination. This has decreased in severity but required ongoing antihistamine use.

No serious adverse events related to vaccination occurred and no prespecified halting rules were met. A 58-year-old individual with an underlying history of hypertension, alcohol use, seasonal allergies, anxiety, and brain aneurysm had a serious unrelated adverse event of upper extremity paresthesias beginning 30 days after bivalent vaccination (**Supplement**).

### Vaccine Immunogenicity

#### SARS-CoV-2 IgG Binding Antibody Geometric Mean Area Under the Curve

**Figure 3:**
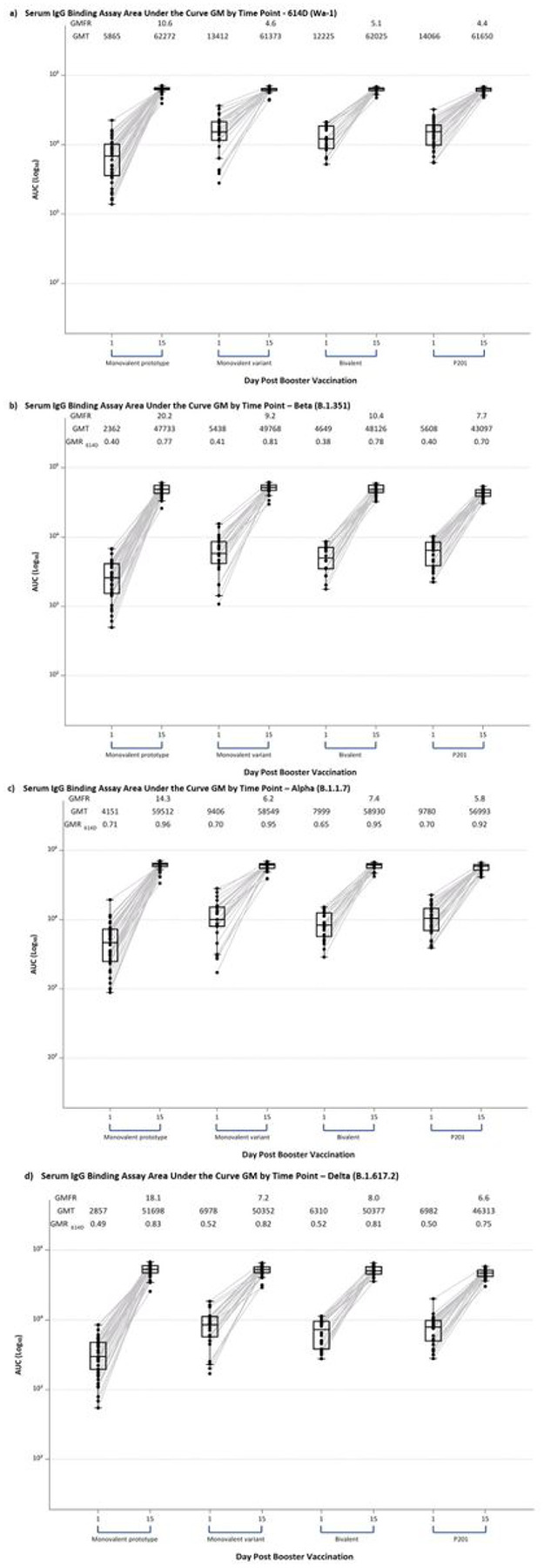
Serum IgG Spike S-2P Binding Area Under the Curve Geometric Mean Titers Distributed by Timepoint and Group. Panel A: 614D, b: Beta (B.1.351), c: Alpha (B.1.1.7); d: Delta (B.1.617.2). Serum IgG Spike S-2P binding antibodies area under the curve before and at 14 days after receiving a third dose of mRNA by study group: Monovalent prototype (100 mcg of mRNA-1273), Monovalent variant (50 mcg of mRNA-1273.351), and Bivalent (25 mcg of mRNA-1273, 25 mcg of mRNA-1273.351). Binding antibodies were observed in all participants for 614D, Beta, Alpha, and Delta. Responses increased substantially by 2 weeks after receipt of a third dose of mRNA vaccine and were similar across the study groups. For comparison, 30 adults that had received an initial 100 mcg of mRNA-1273 in the Phase 2 study followed by a 50 mcg third dose were included (P201). Also, the peak responses observed at 2 or 4 weeks after a second 100 mcg dose of mRNA-1273 in those that had enrolled in the original Phase l study were used for comparison. GMT (geometric mean titer), GMFR (geometric mean fold rise), GMR_614D_ (the ratio of GMTs for the variant of concern divided by the GMT of 614D strain). Boxes and horizontal bars denote interquartile range (IQR) and median respectively. Whisker endpoints are equal to the maximum and minimum values below or above the median +/− 1.5 x IQR.

Prior to the third dose, IgG binding antibodies expressed as geometric mean (GM) of the area under the curve (AUC) to the ancestral 614D (Wa-1) strain S-2P were observed in all participants [monovalent prototype group 5,865 AUC (95% CI 4,711, 7,303); monovalent variant group 13,412 (95% CI 10,064, 17,872); bivalent group 12,225 (95% CI 10,049, 14,873)], indicating binding antibody persistence at a median of 9.5 and 10.6 months following the primary series (Figure 3, **Supplementary Table S9**). Since nearly all participants in the monovalent prototype group received a 25 or 50 mcg primary series (Figure 1), testing was restricted to these participants. Pre-boost S-2P IgG levels correlated with the primary vaccine series dose against all tested variants with numerically higher titers persisting in participants that received higher prime series doses; non-overlapping confidence intervals were only observed in comparison to those that had received a 25 mcg prime series (**Supplementary Table S8**).

Following booster vaccination, a robust anamnestic response to similar titers was observed in all three groups to 614D S-2P at Day 15 [monovalent prototype 62,272 AUC (95% CI 59,973, 64,659); monovalent variant 61,373 (95% CI 58,622, 64,254); bivalent 62,025 (95% CI 59,468, 64,691)] (Figure 3, **Supplementary Table S9**). To provide context, samples from 30 Phase 2 mRNA-1273 study (P201, NCT04405076)^5,15^ participants who had received 100 mcg mRNA-1273 primary series followed by 50 mcg mRNA-1273 boost approximately 6 months later, were also tested, with similar results on Day 15 after the third dose (61,650 (95% CI 59,609, 63,762)).

Pre-boost IgG binding levels to the Beta variant S-2P antigen were approximately 60% lower than against 614D. However, in all groups, the third dose of mRNA vaccine induced similar anamnestic responses against B.1.351 [monovalent prototype 47,733 AUC (95% CI 44,932, 50,710); monovalent variant 49,768 (95% CI 46,282, 53,517); bivalent 48,126 (95% 44,728, 51,781)] that were only 20% lower than the Day 15 responses to 614D (Figure 3, **Supplementary Table S9**). IgG binding for other variants (Alpha, Delta) showed a similar pattern.

#### SARS-CoV-2 Pseudovirus Neutralization ID_50_ Titers

**Figure 4:**
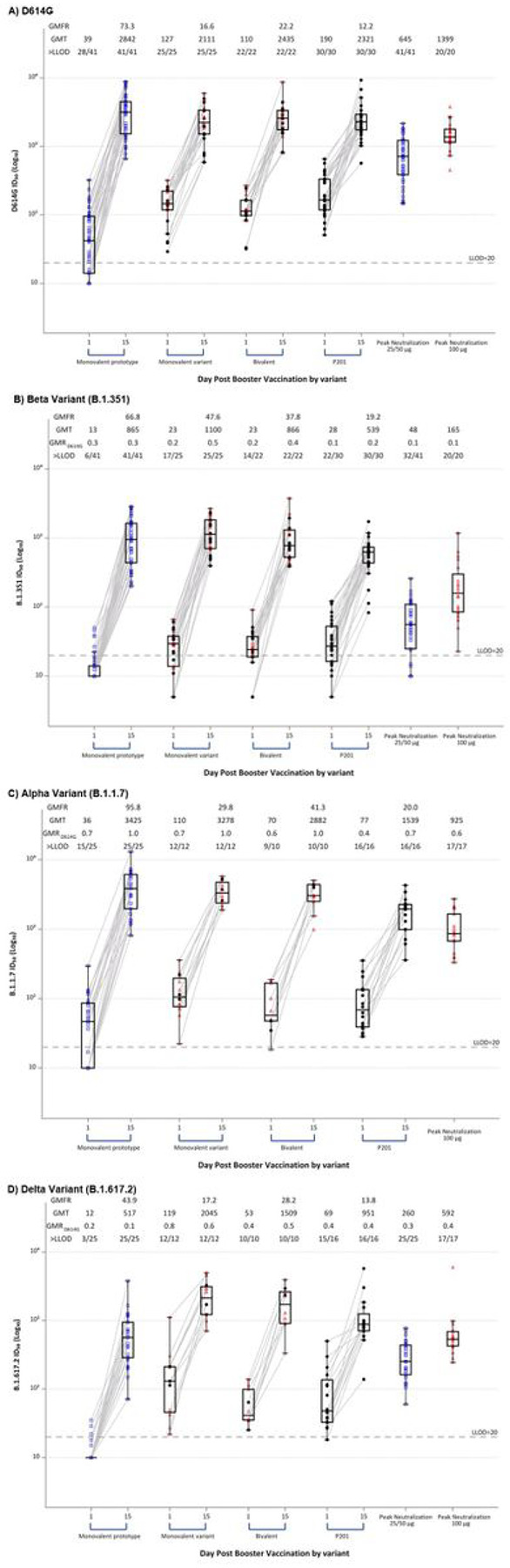
Serum Pseudovirus Neutralization Assay ID_50_ Titers Distributed by Timepoint and by Variant. A) D614G; B) Beta Variant; C) Alpha Variant; D) Delta Variant Serum Pseudovirus Neutralization Assay ID_50_ Titers before and at 14 days after receiving a third dose of mRNA by study group: Monovalent prototype (100 mcg of mRNA-1273), Monovalent variant (50 mcg of mRNA-1273.351), and Bivalent (25 mcg of mRNA-1273, 25 mcg of mRNA-1273.351). Serum Pseudovirus Neutralization Assay ID_50_ Titers were low to undetectable in many participants at baseline, particularly in those who had received 25 or 50 mcg doses of mRNA-1273 for their primary 2 dose series (blue squares in monovalent prototype group), and for Beta and Delta variants. All participants had robust increases in Pseudovirus Neutralization Assay ID_50_ antibody titers by 2 weeks after receipt of vaccine across all the variants. Red triangles denote those that had received an initial 2 dose series of 100 mcg of mRNA-1273. For comparison, titers were similar or greater than those of 30 adults that had received an initial 100 mcg of mRNA-1273 in the Phase 2 study followed by a 50 mcg third dose (P201). Peak pseudovirus neutralization titers observed (at 2 or 4 weeks) after the 100 mcg second dose in the Phase 1 mRNA-1273 participants was provided as a comparator (Peak Neutralization 100 mcg). When available (for D614G, Beta, and Delta variants), peak titers observed (at 2 or 4 weeks) after the 25 or 50 mcg second dose in the Phase 1 mRNA-1273 participants was provided as a comparator (Peak Neutralization 25/50 mcg). Boxes and horizontal bars denote interquartile range (IQR) and median AU, respectively. Whisker endpoints are equal to the maximum and minimum values below or above the median +/− 1.5 x IQR. LLOD is the lower limit of detection of the assay.

The pseudovirus neutralization responses against D614G spike were measured in two laboratories using a similar assay: a fit-for purpose assay for monovalent prototype booster samples and a validated assay for the monovalent variant and bivalent samples. Prior to the third dose, neutralizing activity ID_50_ against pseudovirus expressing the D614G was low in most participants [monovalent prototype GM titer 39 (95% CI 28, 54); monovalent variant 127 (95% CI 96, 168); bivalent 110 (95% CI 84, 143)] (Figure 4, **Supplemental Table S12**). Detectable titers were observed in most, but not all, of those who had received 25 or 50 mcg primary doses but in all 100 or 250 mcg primary doses participants. At 14 days after the third dose, the D614G ID_50_ had increased to high levels across groups [monovalent prototype 2,842 (95% CI 2,270, 3,560); monovalent variant 2,111 (95% CI 1,615, 2,760); bivalent 2,435 (95% CI 1,912, 3,102)]. Post-boost titers were higher than peak neutralizing titers observed following receipt of the second 100 mcg mRNA-1273 dose [1,399 (95% CI 1,111, 1,762)] and were similar to the 50 mcg mRNA-1273 third dose comparator [2,321 (95% CI 1,890, 2,851)].

Pre-boost neutralizing ID_50_ activity against the Beta variant was low to undetectable (Figure 4, **Supplemental Table S11**). Post-boost Beta responses were robust in all groups [monovalent prototype 865, (95% CI 672, 1,113); monovalent variant 1,100 (95% CI 867, 1,395); bivalent 866 (95% CI 659, 1,139); Phase 2 comparison group 539 (95% CI 428, 680)] and exceeded those after the 100 mcg primary series [165 (95% CI 105, 259)].

Pseudovirus neutralizing activity to other SARS-CoV-2 variants was evaluated in a subset of participants (Figure 4, **Supplemental Tables S11–12**). Pre-boost neutralizing titers for variant spike proteins Alpha and Delta were low to non-detectable in most participants that received a 25 or 50 mcg primary series while those that had received a 100 or 250 mcg primary series had more persisting neutralizing activity. Robust serum neutralizing activity against all variants tested was noted post-boost, irrespective of the booster vaccination group, although titers for Delta trended higher after a variant-containing booster [monovalent prototype 517 (95% CI 361, 740); monovalent variant 2,045 (95% CI 1,379, 3,034); bivalent 1,509 (95% CI 885, 2,571); P201 951 (95% CI 618, 1,463)]. Titers were greater than Delta peak titers following the primary 100 mcg series [592 (95% CI 413, 849)].

#### SARS-CoV-2 Focus Reduction Neutralization Test (FRNT) ID_50_ Titers

FRNT ID_50_ live-virus neutralization assays were performed on a random subset of samples in each group. Pre-boost FRNT ID_50_ levels against D614G were low in all participants [monovalent prototype 44 (95% CI 30, 64); monovalent variant 97 (95% CI 58, 161); bivalent 93 (95% CI 46, 187)] (**Supplemental Figure S4, Supplemental Table S16**). FRNT ID_50_ post-boost responses were robust in all groups [monovalent prototype 3,375 (95% CI 2,374, 4,796); monovalent variant 2,829 (95% CI 2,139, 3,740); bivalent 2,392 (95% CI 1,664, 3,438); P201 comparator 2,084 (95% CI 1,564, 2,777)], and were higher than the peak primary series responses [750 (95% CI 549, 1024)].

Pre-boost FRNT titers against Beta were low to undetectable in all groups [monovalent prototype 13 (95% CI 10, 17); monovalent variant 28 (95% CI 17, 48); bivalent 25 (95% CI 13, 48)] (**Supplemental Figure S4, Supplemental Table S16**). Post-boost FRNT responses were robust [monovalent prototype 1,063 (95% CI 717, 1,575); monovalent variant 2,020 (95% CI 1,222, 3,339); bivalent 969 (95% CI 609, 1,540)]; P201 comparison samples 533 (95% CI 360, 788)] and were significantly greater than the peak response observed after the two-dose primary series [163 (95% 93, 286)], but numerically lower than D614G.

#### SARS-CoV-2 CD4+ and CD8+ T Cell Responses

**Figure 5:**
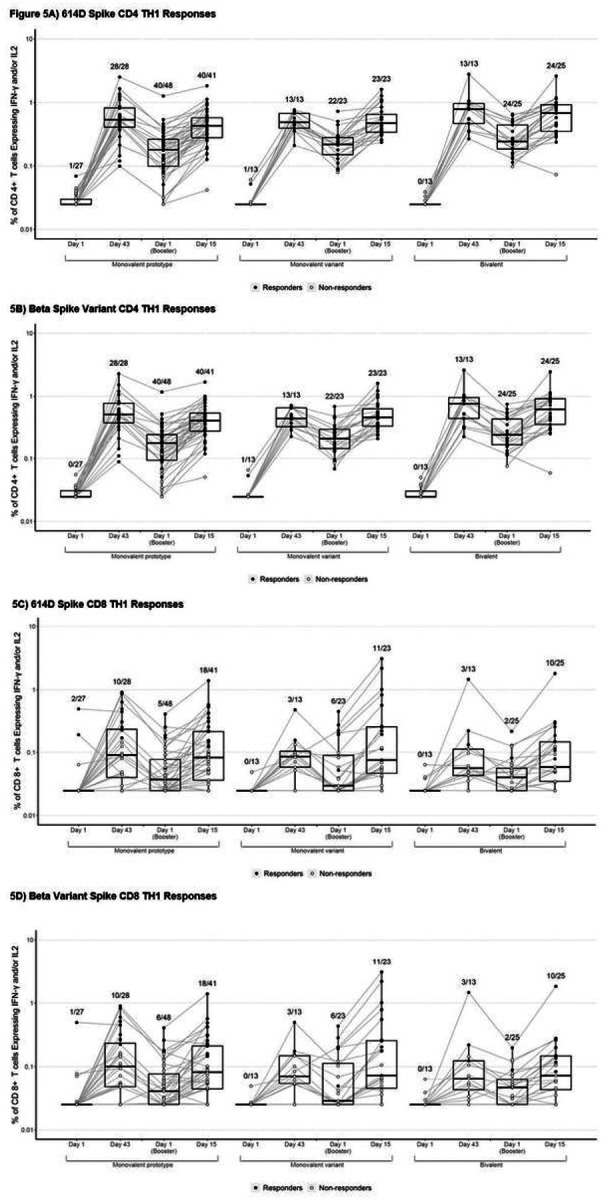
Spike-Specific IFN-g and/or IL-2 T Cell Responses by Group and Timepoint to the A) 614D and the B) Beta Variant SARS-CoV-2 Spike Peptide Pools. Background-adjusted % of CD4 T cells expressing IFN-g and/or IL-2 in response to peptides from the A) 614D spike and B) the beta variant spike, respectively. Background-adjusted % of CD8 T cells expressing IFN-g and/or IL-2 in response to peptides from the C) 614D spike and D) the beta variant spike. Data values below 0.025% are displayed at 0.025%. Closed or open circles indicate whether the sample was deemed a spike-specific T cell “responder” or “non-responder” by Fisher’s exact test, respectively. The numbers on the graph indicate the response rate (i.e. the number of positive responders over the total number tested for each group and timepoint).

A validated intracellular cytokine staining assay detected Th1 and Th2 CD4+ and CD8+ T cells following *ex vivo* stimulation with overlapping 15-mer peptides spanning either prototype (614D) or Beta spike protein sequences. Similar to a previous report,^4^ robust spike-specific IFN-γ and/or IL-2 CD4+ T cell responses were observed at 14 days after the primary series doses (Figure 5; **Supplemental Table S19**). Spike-specific CD4 T cell responses were cross-reactive against the Beta spike with similar magnitudes and response rates compared to the vaccine-matched spike. At the time of boost, spike-specific CD4 T cells declined but remained detectable in nearly all subjects (Figure 5; Supplemental Table S19).

Spike-specific IFN-γ and/or IL-2 CD4+ T cells recognizing both 614D and Beta increased 2.15–2.79 fold following the boost, returning to similar magnitudes (median 0.43–0.68% of CD4 T cells) and response rates (>96%) as observed at 14 days after the primary series (Figure 5, **Supplementary Table S19**). Spike-specific CD4 T cells were polyfunctional with high expression of IFN-g, IL-2, TNF-a, and CD154 (CD40L) (**Supplementary Table S19**). Overall, the Th1 CD4 T cell responses were primarily comprised of effector memory and to a lesser extent central memory cells (**Supplementary Figure S7**). CD4+ T cell Th2 responses, as measured by spike-specific IL-4 expression, were modest (0.01–0.02% of CD4 T cells) and substantially lower than Th1 responses (Figure 5, **Supplementary Figure S6**).

Notably, boosting with the monovalent variant (Beta) mRNA vaccine did not improve cross-reactive responses. Even with the Beta-specific peptides, the variant-recognizing spike-specific CD4 T cell responses were not significantly improved in monovalent variant or bivalent participants (Supplemental **Figures 11 – 12**). However, all three groups tended to have higher spike-specific CD4 T cell responses to prototype-matched as compared to Beta variant peptides.

Spike-specific IFN-γ and/or IL-2 expressing CD8+ T cells were 0.06–0.09% of the total, were primarily effector memory cells, and were detected in 23–36% of subjects at 2 weeks post-2nd vaccination (Figure 5, **Supplementary Table S20**) but then declined. After the boost, spike-specific CD8+ T cells were detected in 40–48% of vaccine recipients with median frequencies of 0.06–0.08%. When spike-specific CD8+ T cells were observed, they cross-reacted against Beta, regardless of group (Figure 5).

## Conclucion

Despite the high efficacy of the mRNA-1273 vaccine, particularly against severe COVID-19 demonstrated in the Phase 3 trial,^1,16^ declines in efficacy over time and against emerging variants have been observed.^13^ Emergence of the Beta variant (1.351), against which sera from mRNA-1273 vaccine recipients had weak cross-neutralization,^11^ spurred development of mRNA-1273.351. Concern over waning immunity, particularly for the participants who received the lowest doses of mRNA-1273 (25 or 50 mcg) in the original Phase 1 study, prompted a decision to amend the protocol to offer a boost of 100 mcg mRNA-1273 to Phase 1 study participants (monovalent prototype). Participants who received doses of 50, 100, or 250 mcg in the Phase 1 study also had the option to join a new trial and be randomized to receive either a monovalent variant or a bivalent booster vaccine. This provided an opportunity to assess humoral and cellular immunological responses after boosting with mRNA-1273, mRNA-1273.351, or bivalent vaccine.

The most common solicited adverse reactions after the third dose of mRNA vaccine were injection site pain, fatigue, myalgia and chills, and the frequency of these events was similar across the three booster vaccine strategies (monovalent prototype, monovalent variant, and bivalent groups). Most adverse reactions were mild or moderate, with severe solicited events occurring in 8 – 13% of participants across the 3 groups. A single participant, who had received an initial two-dose series of 250 mcg, developed a related severe pruritic generalized rash that gradually improved. Additionally, a single participant in the bivalent vaccine group had a transient ischemic attack which was considered unrelated to vaccine at Day 30.

We have previously reported that mRNA-1273 induced binding and neutralizing antibodies against the ancestral 614D strain in participants in the DMID 20–0003 Phase 1 trial that persisted through 180 days after the second 100 mcg dose.^11^ Extending those observations, we found that binding and neutralizing antibodies continued to decline but remained readily detectable at 10–11 months (prior to third dose) in the majority of subjects irrespective of age and initial primary series dose (e.g. 25, 50, 100 and 250 mcg) (**Supplementary Tables S8, S11, S15**). In comparison, pre-boost neutralizing activity against Beta and Delta variants had declined substantially from the peak values observed after the primary 100 mcg series.^9,10^

A robust anamnestic response was observed 14 days after boosting with all 3 vaccines raising serum IgG binding titers and neutralizing activity to levels observed after a 50 mcg boost of mRNA-1273 (EUA booster formulation) (Figure 3, **Supplementary Table S9**). Despite minor differences in post-boost neutralizing antibody titers the overall impression is that boost with prototype, variant monovalent or bivalent vaccines resulted in strong functional antibody responses to all tested variants (Figure 4, **Supplementary Figure S1, Supplementary Table S9**). Notably, post-boost responses exceeded antibody levels achieved 2 weeks after the second primary dose against each respective variant (Figures 3 and 4, **Supplementary Tables S9, S12**, and **S16, Supplementary Figure S4**).

Support for humoral immune responses as a correlate of protection is provided by an assessment of the mRNA-1273 Phase 3 participants, evaluating the correlation between binding and neutralizing antibody levels with risk of symptomatic COVID-19 through 3–4 months post second vaccination.^17^ In that study, subjects with an undetectable pseudovirus neutralization ID_50_ at Day 57 had only 51% protection (95% CI−51 – 83%) against COVID-19. In this study, neutralizing antibodies had waned to this level by the time of the booster in some individuals, particularly those that had received 25 or 50 mcg doses. A third dose of vaccine resulted in D614G GMTs that exceeded 500 for all variants, which exceeds levels that correlate with 90.7% efficacy (95%CI 86.7, 93.6%). While binding and pseudovirus neutralization titers through Day 57 in the Phase 3 trial correlated with protection,^15^ the correlation was incomplete suggesting a potential role of T cells.

A two-dose vaccination regimen with the mRNA-1273 vaccine elicits predominantly Th1-biased CD4+ T cell responses irrespective of age and dosage,^4^ which even in the lower dosage groups (25 and 50 mcg) has been shown to persist through at least 6 months after the second dose.^19^ The T cell data described here highlights the persistence of TH1-biased CD4+ cells through approximately 10–11 months post vaccination, while Th2 CD4+ T cells are less frequently detected and at a lower magnitude.^4^ TH1-biased CD4+ responses after the third dose were similar across the groups and were similar to those observed after the second dose. Effector, and to a lesser extent, central memory CD4+ T cell responses were most robust (**Supplementary Figure S7**). Spike-specific CD8+ T cells were rare at the time of boost but were detected in 40–48% of vaccine recipients after the boost. No particular benefit was observed in the CD4+ or CD8+ responses to the Beta-mutated peptides with use of the monovalent variant or bivalent vaccines. There was significant cross-reactivity in spike-specific CD4 T cell responses detected among all vaccinated subjects, even after the original prototype vaccine series. CD4 T cell responses are sufficiently polyclonal to tolerate the reduction or loss of recognition among the Beta variant epitopes.

Limitations to this study bear consideration. The data represent a small study of non-diverse participants; quantitative comparisons are limited. In the Phase 1 study, participants received 25 – 250 mcg primary series doses. The primary series dose impacted the durability of binding and neutralizing antibodies observed prior to the third dose but did not significantly impact the responses observed post-boost. Participants here received a third dose of mRNA vaccine at a median of 9.5 – 10.6 months after their second vaccination, while booster vaccinations are recommended for adults in the US at 6 months after the primary series. Different mRNA doses were used for the boost (100 mcg in monovalent prototype group, 50 mcg total in monovalent variant and bivalent groups). We partially addressed this by providing a comparison of serological responses observed in 50 mcg mRNA-1273 boosted P201 participants. The mRNA vaccine utilized here in the monovalent variant and bivalent groups was based on the Spike protein of the Beta variant. As such, the ability to extrapolate to vaccines developed in response to other variants (e.g., Delta, Omicron) is limited. Although preliminary data demonstrate that binding and pseudovirus neutralizing antibody titers obtained from the Phase 3 clinical trial of mRNA-1273 correlate with protection, the threshold predictive of protection against emerging variants is unknown.

In conclusion, humoral and cellular immune responses elicited by the mRNA-1273 vaccine persist at least through 10–11 months after the primary series. A third dose of vaccine serves to broadly elevate levels of binding antibodies that recognize the Spike proteins of several variants including Beta and Delta. Booster vaccination with the prototype, variant, and bivalent vaccine strategies all resulted in strong functional neutralizing antibody responses to the prototype and tested variant strains. Similarly, no particular benefit or detriment was observed in the cellular responses to epitopes within the Beta-mutated peptides with use of the monovalent or bivalent Beta variant vaccines. Together these data support the current recommendation for a monovalent prototype mRNA-1273 boost in providing broad immunological cross-protection across variants. However, novel monovalent variant or multivalent mRNA vaccine refinements may lead to improvement in cross-protection and durability and should be considered for further development.

## Methods

### Trial Designs and Participants

Additional details of the design, conduct, oversight, analyses, and results of the trials can be found in the protocols, statistical analysis plans, and Supplement. The 20–0003 substudy (NCT04283461) and the 21–0002 (NCT04785144) trial protocols were reviewed and approved by the Advarra institutional review board. Both protocols permitted interim analyses to inform vaccination strategies and public health. This report includes findings through 28 days after the third mRNA vaccination. The booster vaccination evaluations were conducted at the Kaiser Permanente Washington Health Research Institute in Seattle, the Emory University School of Medicine in Atlanta, and the VRC at National Institutes of Health in Bethesda.

Participants were healthy adults who must have received two doses of mRNA-1273 in the Phase 1 study; other eligibility criteria are included in the protocols (**Supplement**). All third doses of mRNA vaccine were administered between March and April 2021 and recipients will be followed for safety, reactogenicity, and immunogenicity for twelve months after the third dose of mRNA.

Immune responses observed in these monovalent prototype, monovalent variant, and bivalent groups were compared with two other groups that had received an initial two-dose 100 mcg mRNA-1273 primary series: peak neutralization responses after a 100 mcg primary series in 33 participants in the Phase 1 trial (peak pseudoneutralizing antibody titers were also evaluated in those that received a 25 or 50 mcg primary series); and sera from a Phase 2 randomized placebo-controlled trial sponsored by Moderna, protocol P201(NCT04405076)^5,15^ who received a third dose of 50 mcg mRNA-1273 six to eight months after completion of their two-dose 100 mcg mRNA-1273 primary series. These specimens were tested with the same assays in the same laboratories to provide comparisons.

### Trial Procedures

For each protocol, follow-up visits were scheduled for 7, 14, 28, 90, 180, and 365 days after the booster vaccination. Participants recorded local and systemic reactions, using a memory aid, through 7 days after the booster vaccination. Adverse events were graded according to a standard toxicity grading scale (**Supplementary Table 1**).

### Assessment of SARS-CoV-2 Binding Antibody and Neutralizing Responses

For this interim analysis, we tested sera obtained on the day of and prior to the third vaccination through 14 days after this dose. Of the 48 participants in the 20–0003 booster substudy, all but four had received a 25 mcg or 50 mcg dose of mRNA-1273 for the primary series; for consistency, we restricted the assessment of humoral immune responses to participants in those dose groups. For the 21–0002 trial, we tested samples from all participants.

We used a 4-plex Mesoscale Discovery (MSD) platform to detect binding IgG antibodies that has completed validation testing and is undergoing regulatory review.^9,18^ IgG binding against 614D and variants were evaluated using a fit-for-purpose MSD 10-plex assay.^9,18^

Neutralizing antibody responses were assessed by two laboratories using the same SARS-CoV-2 native spike-pseudotyped lentivirus reporter pseudovirus single-round-of-infection neutralization assay (PsVN), and neutralization titers were expressed as the serum inhibitory dilution required to achieve 50% and 80% neutralization (ID_50_ and ID_80_, respectively). A focus-reduction neutralization test (FRNT) assay, which uses live SARS-CoV-2, assessed vaccine-induced neutralizing activity against the D614G, Beta, and other variant strains.^4,19^

### T Cell Analyses

A validated intracellular cytokine staining was used to examine SARS-CoV-2-specific CD4+ and CD8+ T-cell responses using 27-color flow cytometry.^20,21^ Previously cryopreserved specimens were stimulated with four pools of peptides (15 amino acids overlapping by 11 amino acids): conserved S1, conserved S2, beta mutations and original matched, which together covered the spike sequence of the original 614D/Wuhan (Wu-1) and the beta variant. The peptides were pooled according to sequence into the peptides that were conserved between the 614D and beta S1 and S2 subunits, respectively (conserved S1 and conserved S2). The other 2 peptide pools contained just the peptides with mutations in the beta sequence (beta mutations) and a matching set of unmutated peptides (original matched). The interpretation of responses are in the **Supplement**.

## Figures and Tables

**Table 1.** Characteristics of the Participants at Enrollment.*

Vaccine Formulation for the Third Dose	Monovalent Prototype 100 mcg mRNA-1273 N=48	Monovalent Variant 50 mcg mRNA-1273.351 N=25	Bivalent 25 mcg mRNA-1273 + 25 mcg mRNA-1273.351 N=23
**Characteristic**			
Sex — no. (%)			
Male	25 (52)	14 (56)	11 (48)
Female	23 (48)	11 (44)	12 (52)
Age — yr	56.3±16.7	45.0±19.0	43.7±17.0
Age group — no. (%)			
18–55 years	18 (38)	17 (68)	16 (70)
56–70 years	16 (33)	4 (16)	4 (17)
71+ years	14 (29)	4 (16)	3 (13)
Race or ethnic group — no. (%)†			
American Indian or Alaska Native	0	1 (4)	0
Asian	2 (4)	1 (4)	0
Black	0	1 (4)	1 (4)
White	45 (94)	21 (84)	22 (96)
More than one race	1 (2)	0	0
Unknown	0	1 (4)	0
Hispanic or Latino ethnic group — no. (%)	3 (6)	4 (16)	1 (4)
Body-mass index‡	24.4±3.0	24.9±3.3	25.5±3.1
mRNA-1273 vaccine dose primary two dose series — no. (%)			
25 mcg§	29 (60)	0	0
50 mcg	15 (31)	4 (16)	4 (17)
100 mcg	3 (6)	15 (60)	13 (57)
250 mcg	1 (2)	6 (24)	6 (26)
Time interval between second dose of mRNA-1273 and the booster dose — days (months)			
Mean (SD)	294±30 (9.7±1)	321±25 (10.5±0.8)	319±26 (10.5±0.8)
Range	257, 371 (8.4, 12.2)	269, 359 (8.8, 11.8)	262, 359 (8.6, 11.8)
Median	289 (9.5)	324 (10.6)	324 (10.6)
Q1, Q3	267, 317 (8.8, 10.4)	304, 341 (10.0, 11.2)	303, 342 (10.0, 11.2)

* Plus–minus values are means ±SD.

† Race or ethnic group was reported by the participants.

‡ The body-mass index is the weight in kilograms divided by the square of the height in meters. For the homologous boost cohort this calculation was based on the weight and height measured at the time of screening for the 20–0003 protocol, for the heterologous boost groups it was based on weight and height measured at the first visit for the 21–0002 protocol.

§ Participants who received a 25 mcg mRNA-1273 vaccine dose in their primary series were not eligible to receive a mRNA-1273.351 containing 3^rd^ dose.
